# Intracellular biomass flocculation as a key mechanism of rapid bacterial killing by cationic, amphipathic antimicrobial peptides and peptoids

**DOI:** 10.1038/s41598-017-16180-0

**Published:** 2017-12-01

**Authors:** Nathaniel P. Chongsiriwatana, Jennifer S. Lin, Rinki Kapoor, Modi Wetzler, Jennifer A. C. Rea, Maruti K. Didwania, Christopher H. Contag, Annelise E. Barron

**Affiliations:** 1 Department of Chemical and Biological Engineering, Northwestern University, Evanston, Illinois United States; 2 Department of Bioengineering, Stanford University, Stanford, California, United States; 3 Biophysics Program, Stanford University, Stanford, California, United States; 4 Departments of Microbiology and Immunology, Pediatrics, and Radiology, Stanford University, Stanford, California, United States

## Abstract

Many organisms rely on antimicrobial peptides (AMPs) as a first line of defense against pathogens. In general, most AMPs are thought to kill bacteria by binding to and disrupting cell membranes. However, certain AMPs instead appear to inhibit biomacromolecule synthesis, while causing less membrane damage. Despite an unclear understanding of mechanism(s), there is considerable interest in mimicking AMPs with stable, synthetic molecules. Antimicrobial *N*-substituted glycine (peptoid) oligomers (“ampetoids”) are structural, functional and mechanistic analogs of helical, cationic AMPs, which offer broad-spectrum antibacterial activity and better therapeutic potential than peptides. Here, we show through quantitative studies of membrane permeabilization, electron microscopy, and soft X-ray tomography that both AMPs and ampetoids trigger extensive and rapid non-specific aggregation of intracellular biomacromolecules that correlates with microbial death. We present data demonstrating that ampetoids are “fast killers”, which rapidly aggregate bacterial ribosomes *in vitro* and *in vivo*. We suggest intracellular biomass flocculation is a key mechanism of killing for cationic, amphipathic AMPs, which may explain why most AMPs require micromolar concentrations for activity, show significant selectivity for killing bacteria over mammalian cells, and finally, why development of resistance to AMPs is less prevalent than developed resistance to conventional antibiotics.

## Introduction

Antimicrobial peptides (AMPs) are ubiquitous and integral components of innate immunity in virtually every living organism, and are considered promising leads for new antibiotic therapies^1,2^. As a class, AMPs are highly diverse in both primary and secondary structure, which has confounded efforts to elucidate their mechanisms against bacteria. Initially, investigations of their mode of action focused almost entirely on interactions between AMPs and the bacterial cytoplasmic membranes, since many leading researchers in the field hypothesized that membrane permeabilization or disruption could fully explain their activity^3–5^. Although rarely homologous in sequence or structure, AMPs all adopt amphipathic conformations in their active states, with regions rich in cationic or hydrophobic residues. As such, these peptides are well-suited for interactions with the anionic head groups and hydrophobic core of bacterial phospholipid membranes. However, studies of AMPs like buforin II^6^ and indolicidin^7^ have revealed a lack of correlation between membrane permeabilization and antimicrobial activity. Consequent investigations demonstrated that members of this non-membrane-disruptive subset act on intracellular targets, and affect DNA, RNA, and/or protein synthesis^8–11^. Nevertheless, the majority of AMPs remain classified as “membrane-disruptive” with regard to their mode of action.

While many AMPs are both potent and selective antibiotics, the poor bioavailability (i.e. rapid *in vivo* proteolytic degradation) of peptides limits their clinical use primarily to topical applications with only a few AMPs currently being targeted for systemic delivery^12^. Such challenges have spurred the development of non-natural peptidomimetics^13,14^ like peptoids, which are sequence-specific N-substituted glycine oligomers^15^. Peptoids are isomerically related to peptides in that their side chains are appended to the backbone amide nitrogens rather than the α-carbons; as a result, peptoids are not proteolyzed^16^, and offer greater therapeutic potential than peptides. Antimicrobial peptoids (“ampetoids”) have been shown to be analogous to AMPs—the structure-activity relationships that describe the two classes are congruent, suggesting they operate *via* analogous mechanisms^17–20^. Given their similarities, we have investigated the mechanisms of AMP and ampetoids concurrently, studying their interactions with both model membranes and real bacteria.

Recent work investigating the mechanism of action of lysine and tryptophan-rich antimicrobial peptoids^21^ suggests that the killing mechanism involves, to some degree, membrane disruption with probable targeting of intracellular targets such as DNA, RNA, or protein synthesis. Here, we provide further evidence that intracellular targets are integral to the modes of action of AMPs and their non-natural mimics, including those which have previously been assumed to cause bacterial death solely through membrane permeabilization^22,23^. To access the cytoplasm, any molecule must encounter and traverse the plasma membrane; however, membrane activity does not preclude intracellular activity. Based on transmission electron micrographs and soft X-ray tomography of *E. coli* treated with these antibacterials as well as *in vitro* work demonstrating peptoid aggregation of bacterial ribosomes, we hypothesize that the non-specific intracellular interactions of AMPs and ampetoids—driven by both electrostatics and the hydrophobic effect—cause flocculation of polyanions such as ribosomes and DNA. This mechanism causes widespread cytoplasmic disorganization and rapid macromolecular aggregation, leading to a disruption of normal cellular processes and death.

A previously published work on antimicrobial peptoid mechanism of action focused on tryptophan-rich sequences^21^, designed to mimic the AMP indolicidin. In contrast, the work presented herein is focused on ampetoids that are mimics of lysine and phenylalanine-rich AMPs, inspired by the natural AMP magainin-2. Indolicidin and magainin are considered to derive from different families of AMPs based on their amino acid compositions and secondary structures^3,24^. Given the rich diversity of AMPs and their apparent mechanisms of actions, there may likewise be diverse mechanisms of actions among their respective peptoid mimics.

## Results and Discussion

### Membrane permeabilization does not account for antimicrobial activity

**Figure 1:**
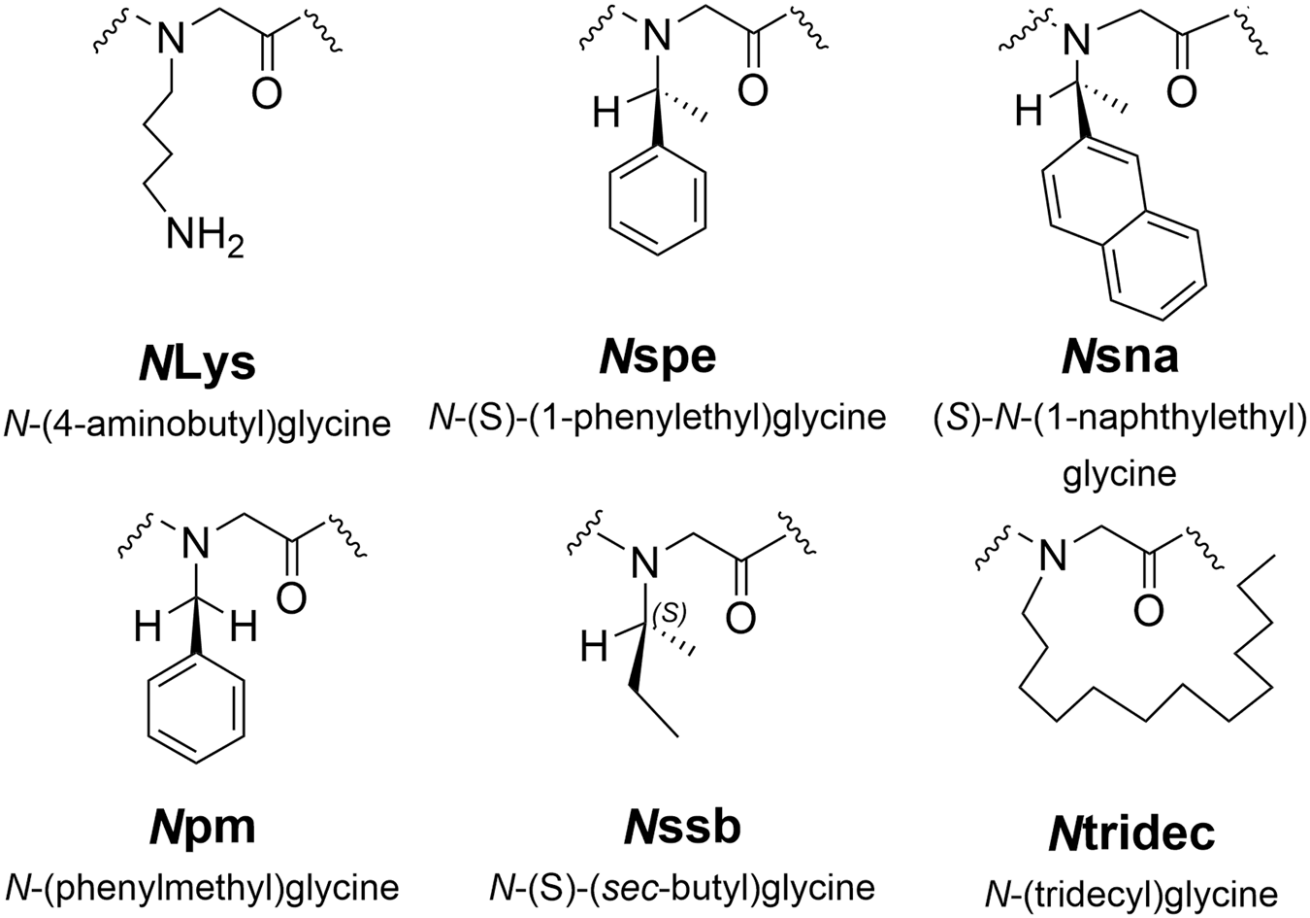
Guide to peptoid monomers.

Because the majority of AMPs are thought to kill bacteria *via* membrane disruption, we used calcein leakage from lipid vesicles, as well as the depolarization of live bacteria, to investigate the relationship between membrane permeabilization and antibacterial activity. The top portion of Table 1 lists the two peptides (pexiganan, an analog of magainin, and bee venom-derived melittin, whose membrane-disruptive activities are well-documented^22,23^) and the peptoids used for these studies, along with their sequences (see Fig. 1 for a guide to peptoid monomers), antibacterial activities against *E. coli* ATCC 35218, and hemolytic activities. These six compounds possess diverse antibacterial and hemolytic activities (pexiganan, peptoid **1**, and **1**-Pro_6_ are selective for bacterial rather than mammalian cells; melittin and **1**_17mer_ are non-selective; and peptoid **2** is an inactive negative control), and are thus well suited for observing trends in permeabilization.

**Table 1:** *In vitro* activities of peptoids and peptides.

Compound	Sequence	% ACN at RP-HPLC elution*	*E. coli* ATCC 35218 MIC (µM)	HD_10_/HD_50_ (µM)
pexiganan	GIGKFLKKAKKFGKAFVKILKK-NH_2_	49.2	3.1–6.3	73/>200
melittin	GIGAVLKVLTTGLPALISWIKRKRQQ-NH_2_	64.3	12.5	1/6
**1**	H-(*N*Lys-*N*spe-*N*spe)_4_-NH_2_	64.2	6.3	14/62
**1**-Pro_6_	H-*N*Lys-*N*spe-*N*spe*-N*Lys-*N*spe-Pro-(*N*Lys-*N*spe-*N*spe)_2_-NH_2_	62.2	12.5	83/>200
**1**-Pro_9_	H-(*N*Lys-*N*spe_2_)_2_-*N*Lys-*N*spe-Pro-(*N*Lys-*N*spe_2_)-NH_2_	62.6	12.5	165 >200
**1**_11mer_	H-(*N*Lys-*N*spe-*N*spe)_3_-*N*Lys-*N*spe-NH_2_	ND	6.3	103/>200
**1**_17mer_	H-*N*spe-*N*spe-(*N*Lys-*N*spe-*N*spe)_**5**_-NH_2_	70.1	25–50	3/15
**2**	H-(*N*Lys-*N*ssb-*N*ssb)_4_-NH_2_	52.4	>100	>200/>200
fowlicidin-1	RVKRVWPLVIRTVIAGYNLYRAIKKK	52.2	1.6	7/>20^†^
LL-37	LLGDFFRKSKEKIGKEFKRIVQRIKDFLRNLVPRTES	63.9	12.5	>100/>100^§^
**1**_achiral_	H-(*N*Lys-*N*pm-*N*pm)_4_-NH_2_	60.4	12.5	180/>200
**1**-*N*Lys_5,11_	H-(*N*Lys-*N*spe-*N*spe-*N*Lys-*N*Lys-*N*spe)_2_-NH_2_	51.2	50	>200/>200
**1**-*N*sna_6,12_	H-(*N*Lys-*N*spe-*N*spe-*N*Lys-*N*spe-*N*sna)_2_-NH_2_	68.1	25–50	7/27
**1**-C13_4mer_	H-*N*tridec-(*N*Lys-*N*spe-*N*spe-*N*Lys)-NH_2_	68.0	12.5	65/>200

*Determined using a gradient of 5–95% acetonitrile (ACN) over 45 minutes, C18 column, 0.2 mL/min; the average of three replicates is reported. ^†^We did not have enough fowlicidin-1 peptide to evaluate hemolysis above 20 µM. ^§^We did not have enough LL-37 peptide to evaluate hemolysis above 100 µM. Note: ND signifies not determined. Values for **1**-Pro_6_ and **1**_11mer_ obtained from ref.^20^. HD_10_/HD_50_ = hemolytic dose; concentration for 10% or 50% hemolysis.

**Figure 2:**
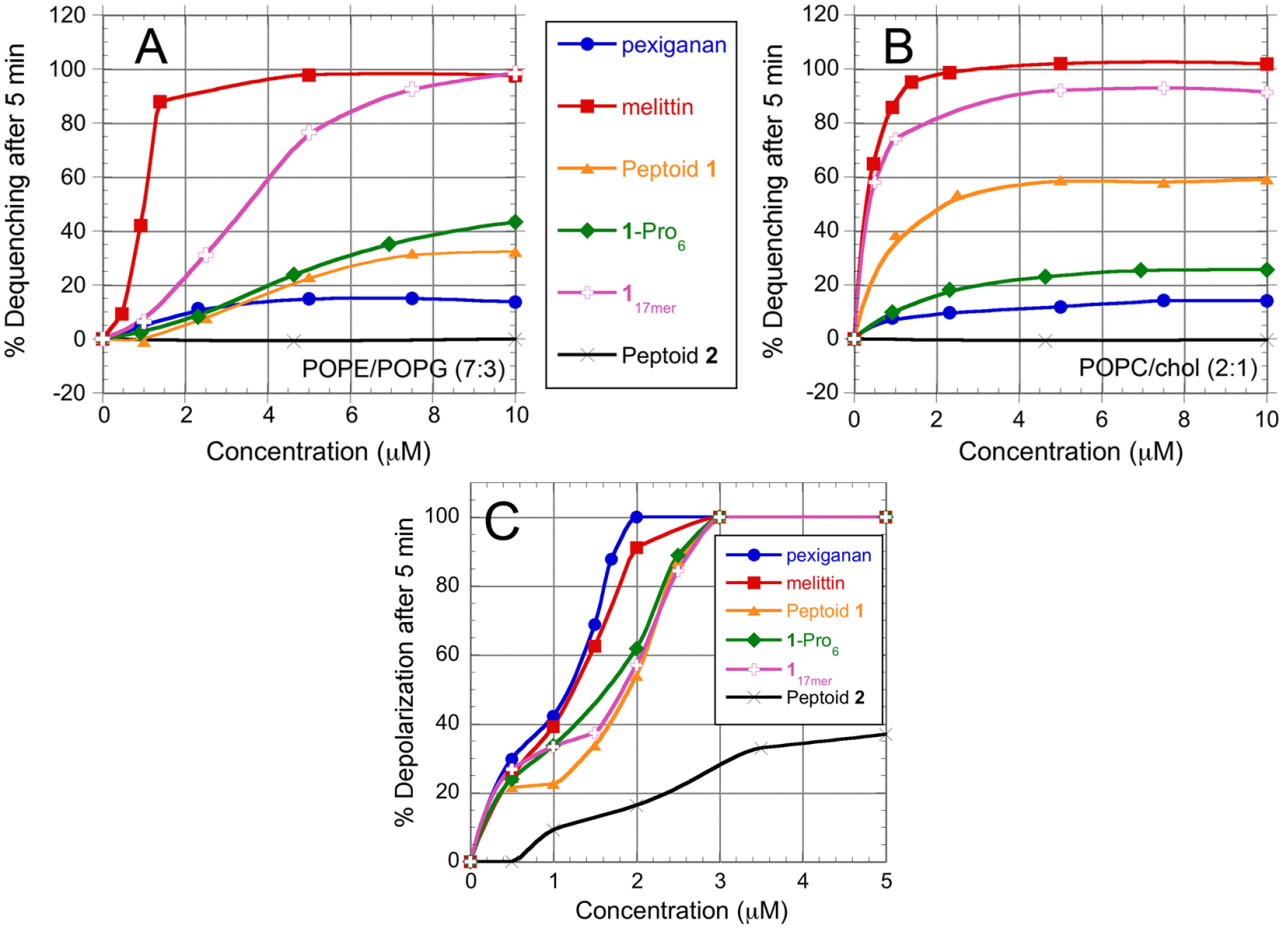
Calcein leakage after 5 minutes from (**A**) anionic, bacteria-mimetic POPE/POPG (7:3) LUVs and (**B**) zwitterionic, erythrocyte-mimetic POPC/cholesterol (2:1) LUVs. (**C**) Depolarization of *E. coli* ATCC 35218 after 5-min. treatment, as monitored by diSC3-5 fluorescence. The data are representative of 3 independent experiments.

Fluorescence detection of calcein leakage from large unilamellar vesicles (LUVs) is a frequently used model system with which to investigate membrane permeabilization by AMPs and ampetoids^9,25,26^. For these studies, two binary lipid compositions were used: (1) anionic, bacteria-mimetic palmitoyloleoylphosphatidylethanolamine (POPE)/palmitoyloleoylphosphatidylglycerol (POPG) (7:3) LUVs (Fig. 2A), and (2) zwitterionic, erythrocyte-mimetic palmitoyloleoylphosphatidylcholine (POPC)/cholesterol (2:1) LUVs (Fig. 2B).

Calcein leakage from POPC/cholesterol LUVs that mimic red blood cell membranes correlated well with hemolytic activity. In contrast, interestingly, there was no overall correlation between bacteria-mimetic POPE/POPG LUV leakage and antibacterial activity, although some of the AMPs and ampetoids strongly permeabilized the anionic membranes. For example, melittin and **1**-Pro_6_ exhibited similar MICs against *E. coli* (Table 1), yet caused very different amounts of leakage from POPE/POPG LUVs (Fig. 2A). Pexiganan, peptoid **1**, and **1**-Pro_6_ were among the most antibacterial compounds in Table 1, and yet caused the least amount of calcein leakage from anionic, bacteria-mimetic vesicles. The incongruity between leakage and antibacterial activity could arise from several factors: (1) lipid vesicles are too simplistic a model for real bacteria, which have a cell wall and, in the case of gram-negatives, an outer membrane, (2) calcein is larger than the molecules whose leakage actually causes death, and (3) the cytoplasmic membrane is not the only target of AMP and ampetoid action. The first two possibilities we addressed by measuring membrane depolarization (in essence, leakage of small ions across the cytoplasmic membrane) in live *E. coli*.

We measured the dissipation of membrane potential in *E. coli* after the addition of antimicrobial peptides or peptoids using the fluorescent potential-sensitive carbocyanine dye diSC3-5^27,28^. As shown in Fig. 2C, depolarization and antibacterial activity corresponded somewhat better than did calcein leakage. Pexiganan, for example, caused the most depolarization at all concentrations tested, and was the most potent antibacterial (Table 1). However, again no overall correlation was observed. Peptoids **1** and **1**_17mer_ depolarized bacteria similarly at concentrations >1.5 µM, yet had significantly different antibacterial activities (Table 1). Furthermore, the concentrations at which maximal depolarization was observed were much lower than the MICs of the compounds. This is especially true in the case of peptoid **1**_17mer_, which had an *E. coli* MIC of 25–50 µM, but caused maximal depolarization at 3 µM against the same bacterial strain.

The observed mismatch between MICs and depolarization might be attributed to our use of 0.2 mM EDTA to facilitate penetration of diSC3-5 through the *E. coli* outer membrane^28^; without EDTA, little or no fluorescence response was observed. However, we do not believe this to be the case, since diSC3-5 experiments on Gram-positive *B. subtilis* (which did not require the use of EDTA) also demonstrated maximal depolarization at concentrations well below the *B. subtilis* MICs (Supp. Fig. S1). Our results agree with reports that show a lack of correlation between bacterial killing and depolarization for many AMPs^27,29^, including polymyxins^28^ analogs of indolicidin^30^, and analogs of melittin containing peptoid residues^31^.

### Changes in surface morphology not always linked to death

**Figure 3:**
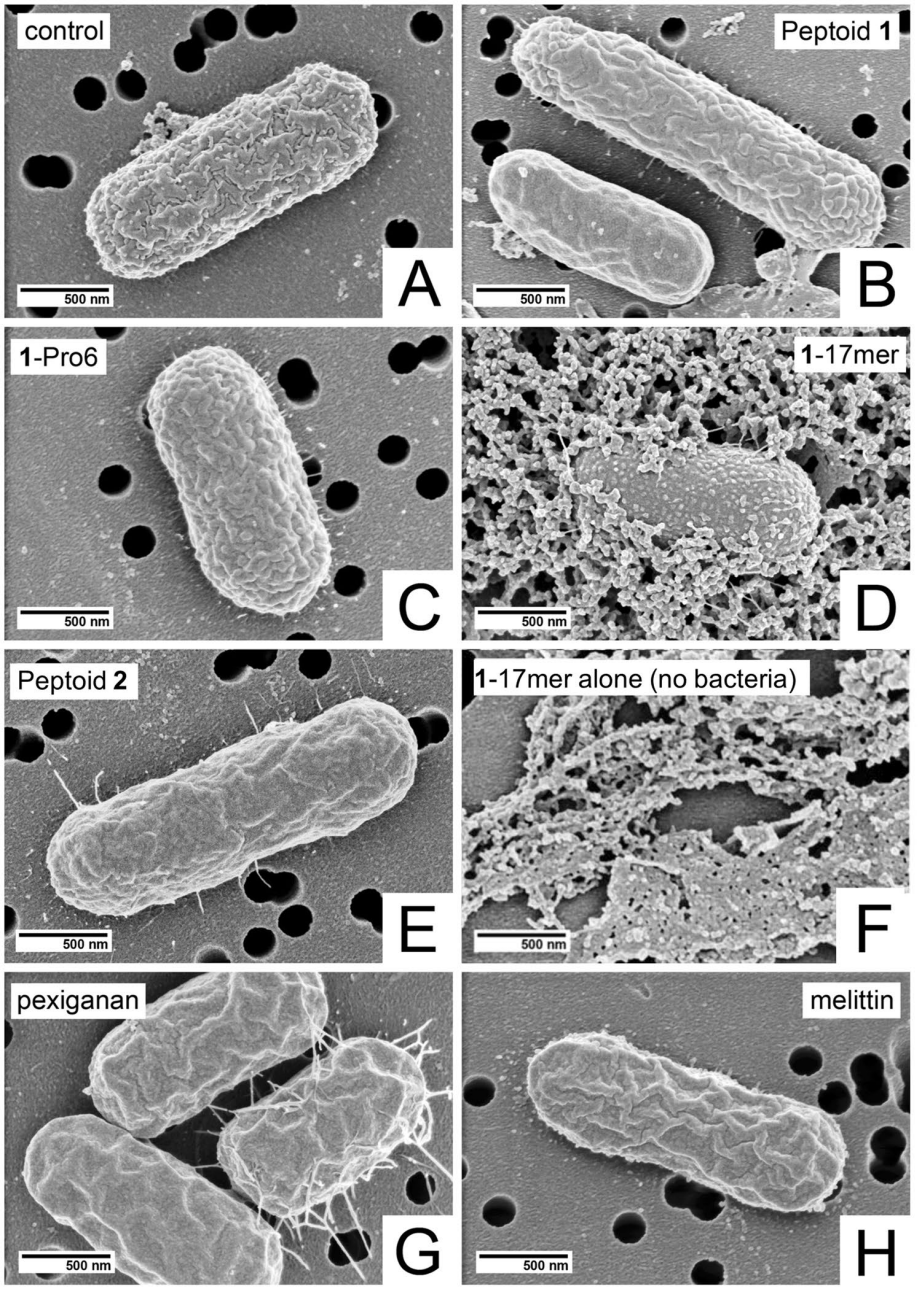
Scanning electron micrographs of *E. coli* either (**A**) without treatment, or treated for one hour with 10 μM (**B**) peptoid 1 (**C**) 1-Pro_6_, (**D**) 1_17mer_, (**E**) peptoid 2, (**F**) 1_17mer_ alone (no bacteria), (**G**) pexiganan, or (**H**) melittin. Magnification = 50,000X.

Because membrane disruption did not fully explain the antibacterial activity of the compounds tested, we next looked at scanning electron microscopy (SEM) to visualize morphological changes on the surface of the bacteria in response to treatment. *E. coli* was treated with 10 μM peptide or peptoid for 1 hour in phosphate-buffered saline (PBS). PBS was used rather than growth media since the use of broth resulted in a large amount of debris, which obscured the surface of the bacteria, making interpretation of the images difficult. We used 10 μM peptide/peptoid for all samples since it is near the MICs of the majority of the oligomers. Bacterial concentrations were adjusted to 5 × 10^5^ CFU/mL, identical to the final concentration of bacteria in MIC assays. Representative micrographs of each sample are shown in Fig. 3.

Before fixation, aliquots of each samples were serially diluted and plated on agar to determine the concentration of live bacteria remaining after the 1-hour treatment. These results are shown in Table 2 and differ from MICs against the same bacterial strains for at least three reasons, as follows. (1) SEM samples were treated in PBS (as discussed above), whereas MIC assays are performed in cation-adjusted Mueller-Hinton broth. AMPs and ampetoids are known to interact with ions in the media (for example, when switching from plain Mueller-Hinton broth to cation-adjusted Mueller-Hinton broth)^1^, so differences in activity may be due to changes in media composition. (2) the treatments were for one hour only, whereas MICs are determined after 16 hours of growth. It may be that bacterial killing by certain compounds does not occur within one hour. (3) Some of these compounds may cause inhibition of growth without causing bacterial cell death (i.e., they are bacteriostatic—which is what the MIC indicates—rather than bactericidal—which is determined by plating and counting colonies). Given these considerations, the bacterial counts in Table 2 are integral to interpreting the SEM images in Fig. 3.

**Table 2:** Bacterial counts following peptoid/peptide treatment for 1 hour and before SEM and TEM sample preparation and DNA retardation assay results. Partially-killed samples are shown in bold.

Compound	SEM	TEM		Retardation assay (µg of compound)*
	Bacterial count after 10 µM treatment (% of control)	Bacterial count after 10 µM treatment (% of control)	Bacterial count after 100 µM treatment (% of control)	
[control]	5 × 10^5^ (100)	1 × 10^8^ (100)	1 × 10^8^ (100)	ND
pexiganan	**2** × **10** ^**1**^ **(0.004)**	**8** × **10** ^**4**^ **(0.08)**	0 (0)	0.6
melittin	**2** × **10** ^**5**^ **(40)**	1 × 10^8^ (100)	**3** × **10** ^**7**^ **(30)**	1.8
fowlicidin-1	ND	**4** × **10** ^**5**^ **(0.4)**	ND^†^	ND^†^
LL-37	ND	**5** × **10** ^**7**^ **(50)**	**4** × **10** ^**6**^ **(4)**	2.0
**1**	**2** × **10** ^**5**^ **(40)**	**2** × **10** ^**5**^ **(0.2)**	0 (0)	3.0
**1**_achiral_	ND	1 × 10^8^ (100)	**3** × **10** ^**4**^ **(0.03)**	1.5
**1**-*N*Lys_5,11_	ND	1 × 10^8^ (100)	**3** × **10** ^**7**^ **(30)**	1.0
**1**-Pro_6_	5 × 10^5^ (100)	1 × 10^8^ (100)	**3** × **10** ^**6**^ **(3)**	7.0
**1**-*N*sna_6,12_	ND	**2** × **10** ^**5**^ **(0.2)**	0 (0)	2.0
**1**_17mer_	**3** × **10** ^**1**^ **(0.006)**	**3** × **10** ^**5**^ **(0.3)**	0 (0)	9.0
**2**	5 × 10^5^ (100)	1 × 10^8^ (100)	**1** × **10** ^**8**^ **(100)**	>145^§^

*The mass of compound required to completely retard migration of 1 µg of DNA. ^†^We did not have enough fowlicidin-1 to evaluate these conditions. ^§^DNA was partially, but not completely, retarded at a peptoid/DNA weight ratio of 145. Note: ND signifies not determined.

In several instances, large external morphological changes occurred upon treatment with peptide or peptoid, yet very few of the bacteria were dead. For example, *E. coli* treated with **1**-Pro_6_ showed a clear “blistering” of the surface, often accompanied by a “thorny halo” on the surface of the substrate (Fig. 3C). This was markedly different from the textured surface of the control (Fig. 3A), yet all of the **1**-Pro_6_-treated *E. coli* were viable after treatment. Treatment of *E. coli* with 10 μM peptoid **2** (Fig. 3E) also yielded a change in surface morphology—a smoothing of the bacterial surface—yet none of the bacteria were dead (consistent with an MIC > 100 μM against the same strain). This smoothing suggests that, despite its low antibacterial and hemolytic activity, peptoid **2** does have some surface activity, also evidenced by its low, but still significant, depolarizing ability (Fig. 2C). However, it is either not potent enough or does not affect the right targets to kill bacteria. Thus, apparently dramatic changes in surface morphology cannot be linked to bacterial death.

Bacteria treated by pexiganan, which were nearly all dead (Table 2 **)**, had a wrinkled, flattened appearance (Fig. 3G). Roughly half of bacteria treated by peptoid **1** (Fig. 3B) and melittin (Fig. 3H) exhibited the blistered morphology while the other half exhibited the wrinkled, flattened morphology. Like melittin-treated *E. coli*, roughly half of peptoid **1**-treated bacteria were dead. Together, these results suggest that the wrinkled, flattened morphology may be correlated with bacterial death, while the blistered morphology precedes, but does not indicate, death.

Peptoid **1**_17mer_ caused swelling of *E. coli* (Fig. 3D), in direct contrast to the wrinkling and/or flattening effected by compounds like pexiganan, melittin, and peptoid **1**. Although the large amount of material surrounding the **1**_17mer_-treated bacterium appears that it may be cytoplasmic material exuded from the cell, control images of 10 μM **1**_17mer_ without bacteria (Fig. 3F) confirm it to be, instead, simply aggregated and precipitated peptoid.

### Marked changes in intracellular morphology

Because neither membrane disruption nor surface morphology could fully explain the relative antibacterial activities of the compounds tested, we used transmission electron microscopy (TEM) to look instead for intracellular changes in *E. coli* morphology after the addition of peptides or peptoids. To expand the range and diversity of sequences tested, we added two peptides (the chicken cathelicidin fowlicidin-1^32^ and the human cathelicidin LL-37^33^) and three peptoids (**1**_achiral_, **1**-*N*Lys_5,11_, and **1**-*N*sna_6,12_) to the panel of compounds used for permeabilization studies (Table 1). Like pexiganan and melittin, the AMPs fowlicidin-1 and LL-37 are currently believed to kill bacteria *via* membrane permeabilization and disruption^22,23,32–38^. Peptoids **1**_achiral_ and **1**-*N*sna_6,12_ add two additional peptoid monomer sequences (Fig. 1, *N*pm and *N*sna, respectively) to the library. In **1**_achiral_, *N*pm is substituted for the chiral, aromatic *N*spe side chains. In **1**-*N*sna_6,12_, two of the phenylalanine-like *N*spe residues are replaced with the bulkier and more hydrophobic *N*sna side chains, yielding a compound with weak antibacterial activity that was quite hemolytic. In **1**-*N*Lys_5,11_, two of the *N*spe residues are instead replaced by *N*Lys, yielding a compound that had weak antibacterial activity but was not hemolytic even at high doses.

**Figure 4:**
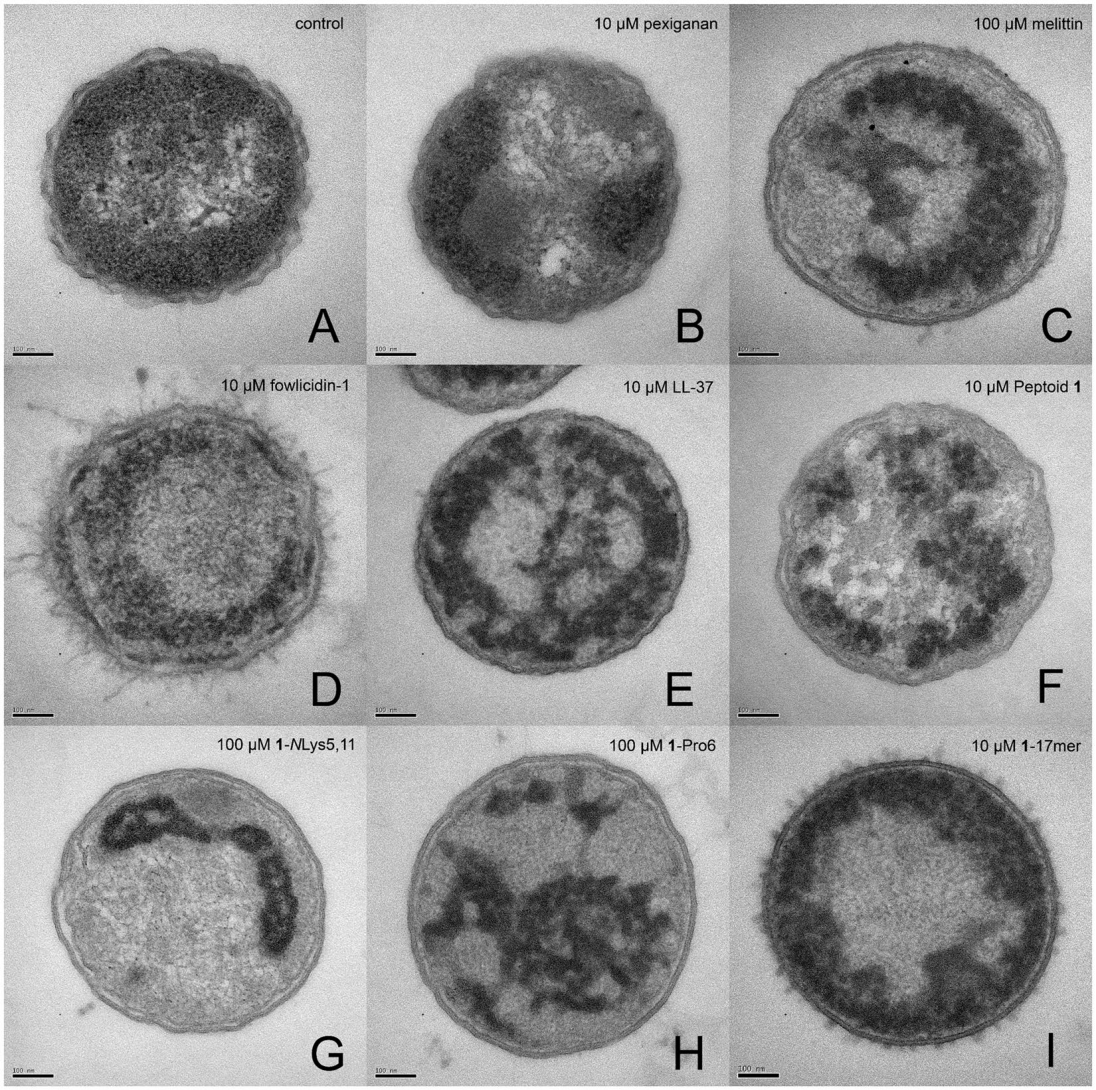
Comparison of transmission electron micrographs of transverse thin sections of representative *E. coli* treated for 1 hour with enough peptide or peptoid to kill some, but not all of the bacteria in the sample. (**A**) No treatment, (**B**) 10 µM pexiganan, (**C**) 100 µM melittin, (**D**) 10 µM fowlicidin-1, (**E**) 10 µM LL-37, (**F**) 10 µM peptoid 1, (**G**) 100 µM 1-*N*Lys_5,11_, (**H**) 100 µM 1-Pro_6_, (**I**) 10 µM 1_17mer_. Images of bacteria treated with 2, 1_achiral_, and 1-*N*sna_6,12_, are shown in Supp. Fig. S2. Scale bar represents 100 nm.

For TEM, the bacteria were treated at peptide/peptoid concentrations that killed some, but not all of the bacteria in each sample after 1 hour (10 µM for some, 100 µM for others) (Table 2) with the exception of inactive peptoid **2**, a strategy used to capture images relevant to the mechanisms of death. Bacteria had to be used at a considerably higher concentration for TEM sample preparation (1 × 10^8^ CFU/mL) than for MIC assays (5 × 10^5^ CFU/mL); this explains why, in some cases, we used peptide/peptoid concentrations well above the MICs of most of the compounds. Because of several discrepancies in TEM and antibacterial assay protocols (i.e. bacterial concentration, time of treatment, etc.), and the possibility that, under the conditions used, some of the compounds are bacteriostatic rather than bactericidal, it was not possible to directly compare MICs with TEM results. To circumvent this, we correlated the observed morphological changes with bacterial death by plating serial dilutions of the treated bacteria immediately before they were enrobed in agarose and prepared for TEM. These bacterial counts are shown in Table 2. Electron micrographs of transverse sections are shown in Fig. 4. Ribosomes appear as numerous, small, electron-dense spots which fill most of the cell; the nucleoid is represented by the centralized electrolucent (apparently “bright”) regions of the cell; and lipid bilayers appear as very thin, dark, parallel lines, about 3 nm apart.

Micrographs of untreated control bacteria (Fig. 4A) exhibited all of the expected components of thin-sectioned *E. coli*—ribosome-rich cytoplasm surrounding the DNA-containing nucleoid, and both inner and outer membranes. The outer membrane had a ruffled appearance, consistent with SEM images we generated of the same strain (Fig. 3A) and atomic force microscopy (AFM) images of the surface of untreated *E. coli* ^39^. The same cellular components were all visible in *E. coli* treated with peptoid **2** (Supp. Fig. S2A) in that no depletion of ribosomes, appearance of large, amorphous, electron-dense regions in the cytoplasm, abolition of DNA aggregation, or smoothing of the outer membrane was observed. The similarities between peptoid **2**-treated bacteria and controls are readily explained by the low antimicrobial potency (Tables 1 and 2) and relatively low membrane activity (Fig. 2) of peptoid **2**, suggesting it disrupts neither membranes nor intracellular components. All other compounds effected large changes in intracellular morphology (Fig. 4B–I), and many did so while leaving both inner and outer membranes apparently intact (Fig. 4C,G,H and Supp. Fig. S2B). These oligomers’ ability to cause cytoplasmic disruption without visibly degrading either membrane explains why no overall correlation was observed between permeabilization or dye leakage and antibacterial activity (Fig. 2, Table 1). Furthermore, none of the compounds induced outright lysis of the bacteria at the concentrations tested—even at concentrations as high as 2 mM, pexiganan, melittin, and **1**_17mer_ were not lytic (Supp. Fig. S3). This result corroborates reports rejecting the classical view that some AMPs, including melittin, kill cells *via* osmotically induced lysis as a result of their permeabilizing actions^40,41^. It should also be noted that most of the compounds (melittin, LL-37, peptoid **1**, **1**_achiral_, **1**-*N*Lys_5,11_, **1**-Pro_6_, **1**-*N*sna_6,12_, and **1**_17mer_) decreased or altogether abolished the ruffled appearance of the outer membrane; however, this “smoothing” of the *E. coli* did not seem related to death, since bacteria killed by 10 µM pexiganan remained ruffled (Fig. 4B). Interestingly, an increase in surface roughness has been observed via AFM for *E. coli* treated with below MIC or at MIC concentrations of the AMPs BP100 and pepR^39^.

Specifically, the appearance of the cytoplasm was altered by all compounds (except the inactive compound, peptoid **2**) in one or more of the following ways: (1) ribosomes were depleted from the cytoplasm, (2) the condensation of DNA, visible in the control, was diminished or abolished, and (3) the native organization of the intracellular compartment, with a central nucleoid surrounded by ribosome-rich cytoplasm, was altered by the appearance of asymmetrical regions of differing electron densities than in the control. For example, treatment with pexiganan caused clear sequestration of the electron-dense ribosomes toward the membrane (Fig. 4B), with areas of cytoplasm depleted of ribosomes. This suggests that the electron-dense aggregates seen in other samples also comprise ribosomes (Fig. 4C–H). It is not surprising that cationic AMPs and ampetoids should be able to cause aggregation of ribosomes, which are polyanionic^42^, due to the favorable electrostatic potential and amphipathic hydrophobicity that mediates their interactions.

The micrographs in Fig. 4 also suggest that cationic AMPs and ampetoids interact with DNA, another ubiquitous polyanion—all compounds except inactive peptoid **2** either attenuated or abolished the dense aggregation of DNA seen in the control (Fig. 4A). For example, DNA condensation was absent from bacteria treated with melittin, fowlicidin-1, LL-37, **1**_achiral_, **1**-Pro_6_, **1**-*N*sna_6,12_, and **1**_17mer_ (Fig. 4C–E,H,I and Supp. Fig. S2B,C). The DNA condensation seen in the control is an artifact well known to be caused by acetone dehydration during sample preparation, since nucleic acids are not fixed by aldehydes or osmium tetroxide^43,44^. Although this can be avoided using *en bloc* uranyl acetate postfixation^45^, we chose not to do so, since the absence of aggregation in some samples suggests the DNA was “fixed” or crosslinked into a structure that resisted condensation upon dehydration.

**Figure 5:**
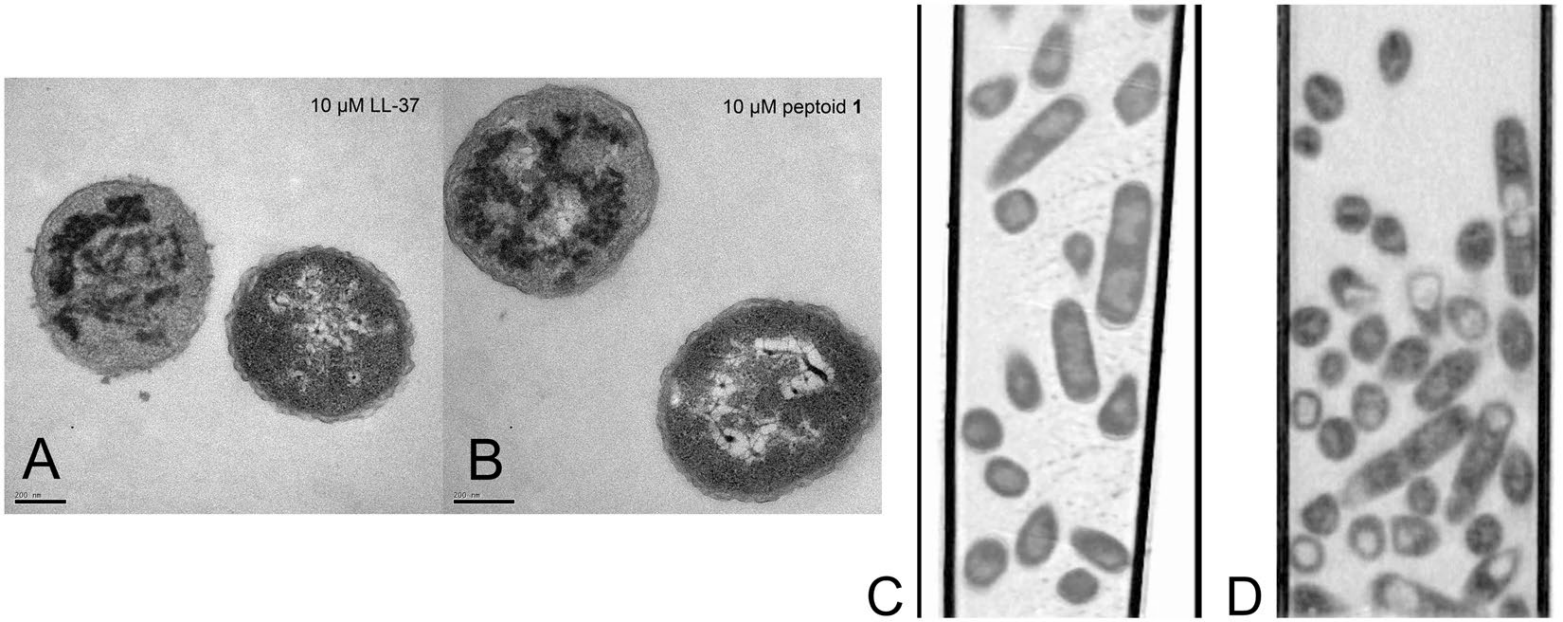
Transmission electron micrographs demonstrating the co-incidence of altered and control-like morphologies in partially killed samples treated with (**A**) 10 µM LL-37 and (**B**) 10 µM peptoid 1. Soft x-ray tomography of *E. coli*. (**C**) Control (untreated) and (**D**) treated with 10 µM peptoid 1. Scale bar (TEM) represents 100 nm.

**Figure 6:**
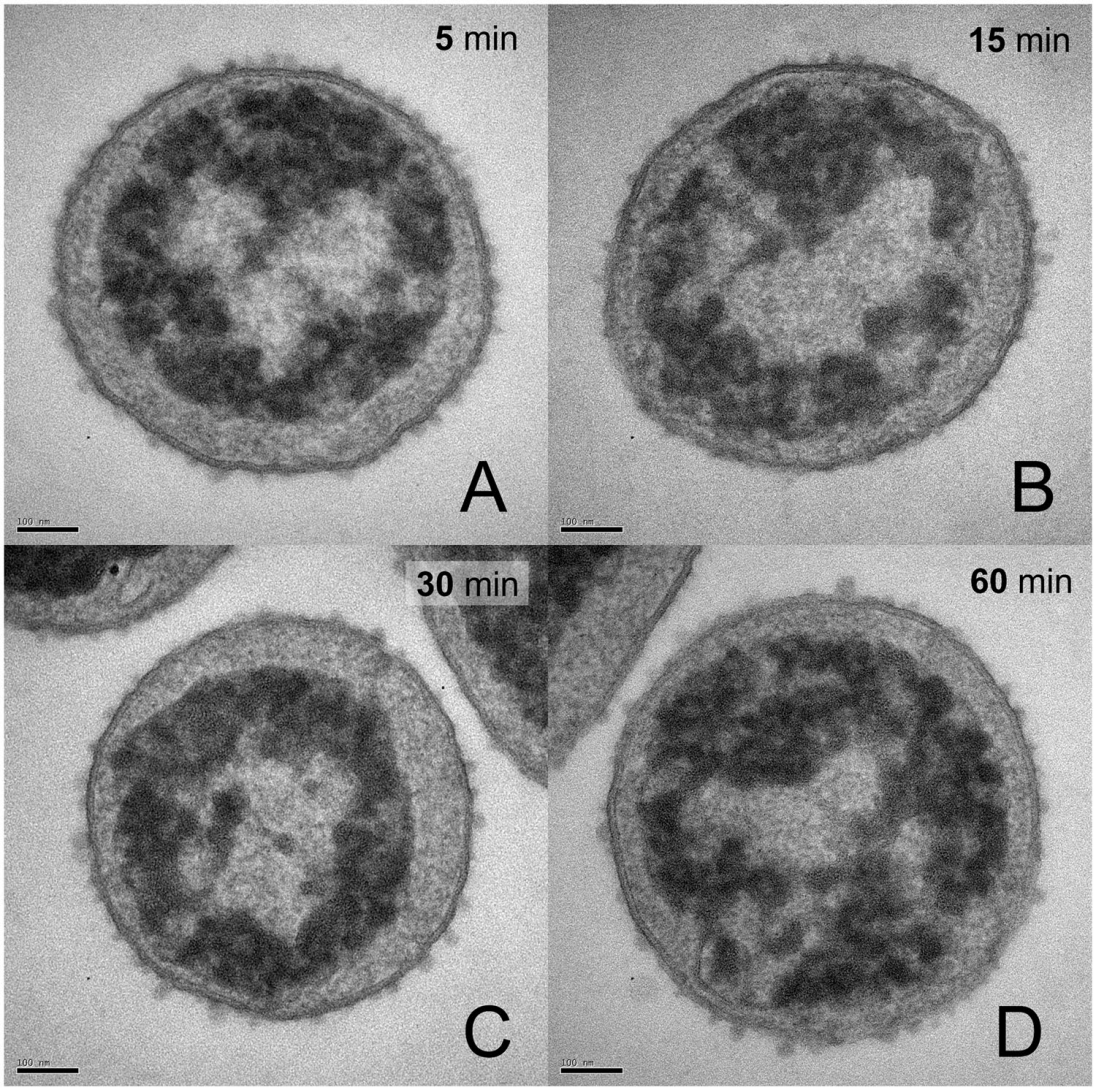
Transmission electron micrographs of *E. coli* treated with 100 µM peptoid 1 for (**A**) 5 minutes, (**B**) 15 minutes, (**C**) 30 minutes, and (**D**) 1 hour. 1 × 10^4^ CFU/mL bacteria remained (0.01% of bacteria in the control) after 5-min treatment; all bacteria were killed after 15-min, 30-min, and 60-min treatments. Scale bar represents 100 nm.

Although it is possible that the observed intracellular morphologies were symptomatic—rather than directly causative—of bacterial death, we believe they reflect the antibacterial mechanism of AMPs/ampetoids for the following reasons. (1) We treated bacteria with partially lethal concentrations of compound. Similar to the way in which the presence of both solid and liquid defines the temperature at the melting point, the co-incidence of live and dead bacteria means we observed them at the transition from viability to non-viability. (2) In cases with a significant percentage ( > ~10%) of live bacteria remaining after treatment, we observed a corresponding mixture of altered and control-like morphologies in the same sample (Fig. 5A,B); thus, the observed morphologies are relevant to the mechanisms of death. (3) Bacteria treated with sub-lethal concentrations of active compound (no bacteria were killed by 10 µM melittin, **1**_achiral_, **1**-*N*Lys_5,11_, and **1**-Pro_6_) were indistinguishable from the control (Supp. Fig. S4), which suggests that altered intracellular morphologies only appeared concomitant with, and did not precede, bacterial death. (4) Bacteria untreated (Fig. 5C) or treated with 10 μM peptoid 1 for 60 minutes (Fig. 5D), flash frozen, and then analyzed using soft X-ray tomography^46,47^ showed changes in the internal biomass distribution remarkably similar to the differences seen between untreated and treated TEM samples, demonstrating that these observed changes are not an artifact of TEM sample preparation. (5) Bacteria treated with peptoid **1** for as little as 5 minutes exhibited significant intracellular morphological changes, similar to those seen after 60 minutes (Fig. 6). The similarity of these four samples demonstrate that the observed changes in morphology are not a post-mortem artifact; rather, they occurred within 5 minutes of treatment, and are correlated with death even after such a short exposure. (6) Studies using fluorescence microscopy and immuno-gold staining followed by TEM, demonstrated that AMPs, including magainin-2 (the peptide from which pexiganan was derived) and LL-37, can penetrate past the inner membrane and reside in the cytoplasm of bacteria^48–51^. (7) Intracellular granulation has been observed by TEM upon treatment of *Clostridium perfringens* with the chicken β-defensin, gallinacin-6 and, most recently, upon treatment of *E. coli* with another chicken AMP, CATH-2^52,53^. This suggests the morphological changes we observed can be ascribed to direct interaction of cytoplasmic species with the AMPs and ampetoids, rather than to indirect effects of membrane permeabilization on the intracellular compartment.

Overall, the observed morphologies, together with numerous reports that AMPs can translocate past the inner membrane and into the cytoplasm, strongly suggest that AMPs and ampetoids interact with intracellular polyanions like nucleic acids and ribosomes, causing them to aggregate or associate in non-native ways. Such behavior is likely due to the electrostatically driven adsorption of AMPs or ampetoids to the electronegative ribosomal surface. As a result, the charge repulsion that normally exists between ribosomes (as well as AMPs/ampetoids) is attenuated or neutralized, and the remaining hydrophobic regions in the peptides/peptoids coalesce with hydrophobic ribosome moieties *via* the hydrophobic effect. Interactions with DNA are likely similar, with AMPs simultaneously interacting with both the anionic DNA strands and the hydrophobic regions on other AMPs or on the DNA bases, effectively creating a crosslinked structure. These phenomena probably do not rely on hydrogen bonding interactions, since peptoids lack backbone hydrogen bond donors, and the effects of AMPs and ampetoids are similar.

Peptoids **1**_17mer_ and **1**-*N*sna_6,12_ effected distinct morphologies compared with the other compounds. Treatment with partially lethal concentrations of these peptoids caused electron-dense regions to appear immediately adjacent to the plasma membranes (Fig. 4I), apparently radiating inwards. At higher concentrations of 100 µM (Supp. Fig. S5), 1-*N*sna_6,12_- and **1**_17mer_-treated bacteria appeared almost completely homogeneous. Given their relatively high hydrophobicities (as measured by RP-HPLC elution, Table 1), it may be that these two peptoids employ an empirically different surfactant-like mechanism of action. Driven primarily by hydrophobic interactions, these peptoids may cause global unfolding and/or disassembly of protein complexes regardless of charge, rather than the aggregation and segregation seen in response to the other compounds.

In general, the resulting morphologies in Fig. 4 were diverse, even when comparing compounds similar in sequence and/or activity. For example, **1**, **1**-*N*Lys_5,11_, and **1**-*N*sna_6,12_ all have closely related sequences (Table 1), but caused very different changes to the membranes and cytoplasm of *E. coli*. This diversity implies that each oligomer interacts with intracellular components in a unique way; indeed, closely related analogs of buforin II have been found to have varying affinities for DNA^54^. Thus, certain AMPs or ampetoids might have a greater affinity for nucleic acids, whereas others might bind and aggregate ribosomes more efficiently. Together with the observation that AMPs occur in nature as mixtures^5^, this may explain the low prevalence of bacteria which have become resistant to AMPs^55^, since a diversity of effects applies inconsistent evolutionary selective pressure.

### DNA retardation assays

By TEM, many of the compounds appeared to interact with the nucleoids of *E. coli*; thus, we used a DNA retardation assay to quantify their affinities for genetic material. Such a method has previously been used to investigate DNA binding of other cationic peptoid sequences^56,57^. The results, shown in Table 2, demonstrate that all compounds except the inactive peptoid **2** were able to retard the electrophoretic migration of DNA plasmids at weight ratios less than ten, confirming the ability of these AMPs and ampetoids to interact with DNA at relevant concentrations. That LL-37 interacts with DNA is not surprising given previous work showing LL-37-mediated DNA delivery into cells^38,58,59^. Other antibacterial sequences found in nature to also be efficient at DNA condensation include pepR, a cationic, amphipathic, α-helical peptide derived from the dengue virus capsid protein^39,60^. Overall, though, there was no correlation between DNA affinity and antibacterial activity. For example, **1**_17mer_ and **1**-*N*sna_6,12_ had comparable antibacterial activities (Table 1), but they had significantly different affinities for DNA. Conversely, pexiganan and **1**-*N*Lys_5,11_ retarded DNA migration at similar ratios, but had very different MICs against bacteria. This lack of correlation is likely due to the wide variety of polyanionic species other than DNA that reside in the cytoplasm, all of which are potential targets for AMPs and ampetoids.

### Kinetics of antibacterial activity

**Figure 7:**
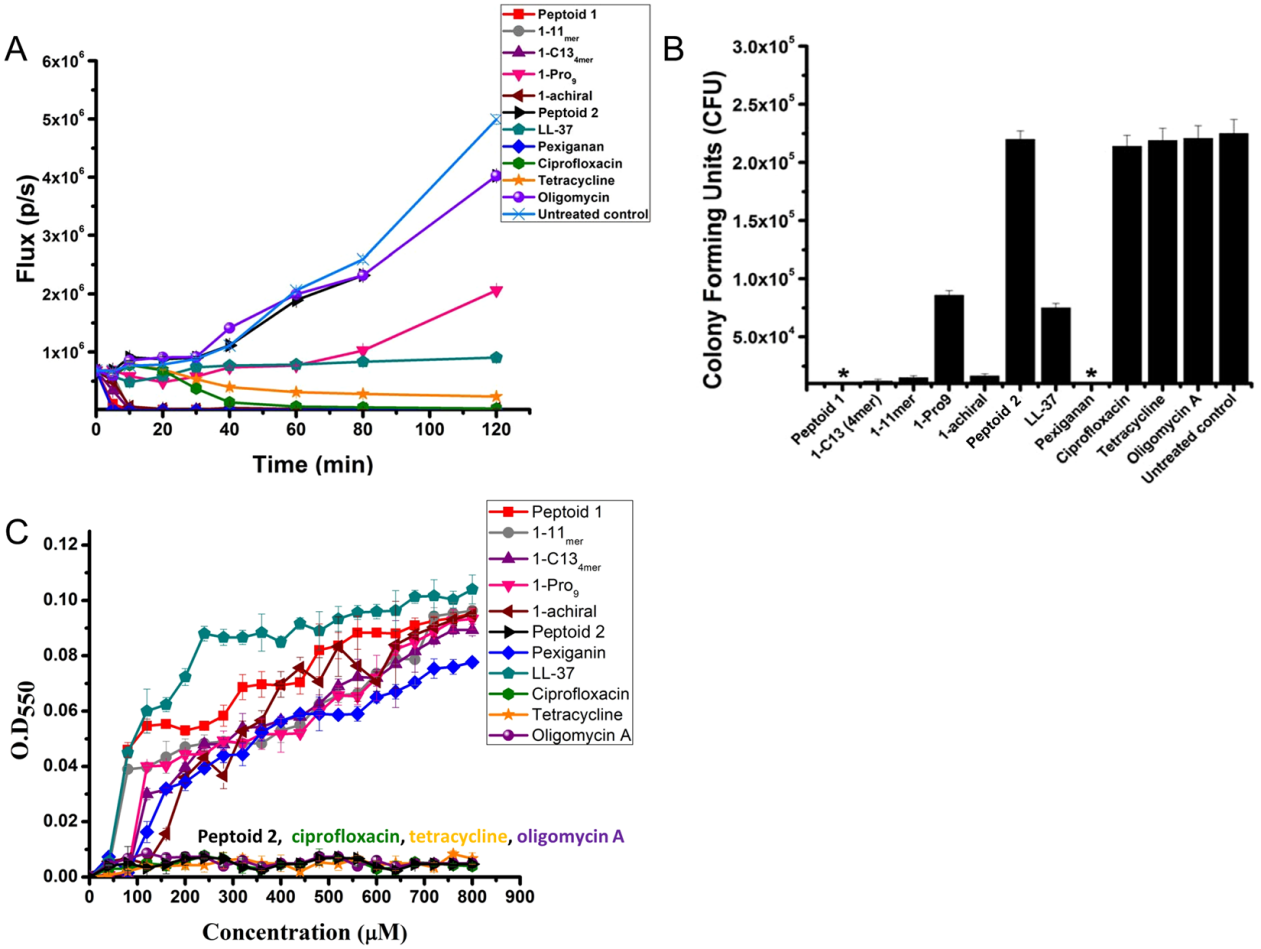
(**A**) Kinetics of antibacterial activity of antimicrobial peptoids, AMPs, and conventional antibiotics against bioluminescent *P.aeruginosa* at 12.5 μM in LB at 37 °C. Reported values are average of three independent experiments with two replicates each. (**B**) Bacterial colony forming units of *P. aeruginosa* after treatment with antimicrobials at 12.5 μM for 10 min in LB at 37 °C. Note: *Represents no colonies were formed on the LB plate. (**C**) Spectrophotometric measure of optical densities of 1 μM bacterial ribosome solution with antimicrobials at λ = 550 nm. Reported values are average of three independent experiments with three replicates each. Note: the spectra are net spectrum = spectrum of antimicrobial + ribosomes − spectrum of antimicrobial. Error bars are represented as mean ± Standard Deviation (SD).

To further explore the mechanism of action, we studied the killing kinetics of AMPs, ampetoids, and conventional antibiotics against luciferase-expressing *Pseudomonas aeruginosa*, another Gram-negative bacterium, against which these peptoids have previously proven to be effective^61^. The decrease in bioluminescence at 37 °C was measured for a range of concentrations from 0 – 100 μM (Supp. Figs. S6 and S7). Kinetic comparisons among the different compounds were made at the previously published lower range of the MIC (12.5 μM) (Fig. 7A). Ciprofloxacin and tetracycline showed a ~97% decrease in bioluminescence at 1 and 2 h, respectively; whereas oligomycin A was inactive at 12.5 μM. Pexiganan showed ~99.9% decrease in bioluminescence within 10 min, while LL-37 caused a ~15% decrease in the bioluminescence within that time. Peptoids **1**, **1**_11mer_, **1**-C13_4mer_, and **1**_achiral_ showed ~99.9% decrease in bioluminescence within 10 minutes while the inactive control, peptoid **2**, was completely ineffective. Compared to an untreated bacterial sample, **1**-Pro_9_ caused ~20% reduction in bioluminescence at 12.5 μM within 2 hours. The decrease in bioluminescence was correlated to bacterial death by measuring the colony forming units (CFU) by plating bacteria on LB agar plates (Fig. 7B) after treatment with antimicrobials at 12.5 μM for 10 min. Compared to no treatment, ciprofloxacin, tetracycline, and oligomycin A did not kill bacteria within 10 minutes. Pexiganan and LL-37 reduced bacterial viability by 100% and ~65%, respectively. Peptoids were active in killing the bacteria within 10 minutes. It is clear that peptoids and AMPs differ in their mechanisms of action from the tested conventional antibiotics since they killed bacteria within 10 min, whereas the conventional antibiotics showed killing after 1 hour. That these peptoids are “fast killers” lends further support to the notion that the intracellular changes in morphology seen by TEM as early as 5 minutes of treatment (Fig. 6) are correlated with cell death.

### Aggregation of bacterial ribosomes

Since peptoids have been shown to interact with DNA (Table 2), we spectrophotometrically tested whether peptoids can aggregate freshly isolated *E. coli* bacterial ribosomes by measuring scattered light at λ = 550 nm (Fig. 7C). Ciprofloxacin, tetracycline, and oligomycin A showed no aggregation of ribosomes up to 800 μM, while pexiganan and LL-37 aggregated ribosomes, increasing the optical density (OD) to 0.07 and 0.10, respectively. Peptoids **1**, **1**_11mer_, **1**-C13_4mer_, **1**_achiral_, and **1**-Pro_9_ caused aggregation of bacterial ribosomes and increased the OD to 0.09 whereas the inactive peptoid **2** did not cause aggregation.

Although many antibiotics are known to bind to bacterial ribosome subunits, they are believed to exert their antibacterial activity by binding to ribosome subunits in a site-specific manner, which peptoids are not expected to duplicate. As expected, we did not observe aggregation of bacterial ribosomes when treated with tetracycline as the antibiotic is known to function by inhibiting protein synthesis by binding to the 30 S ribosome subunit, and not by ribosomal aggregation. Peptoids and peptides caused aggregation of 40X diluted ribosomes in a concentration range of 40–800 μM, whereas the MIC of peptoids is in the range of 12.5–50 μM^61^. This difference in concentration most likely occurs because a fast-growing bacterial cell has a ribosomal concentration of about 40 μM^62^, but since it is difficult to purify and maintain such a high concentration, as well as for convenience, 1 μM ribosomal concentration was used. This 40X *in vitro* vs. *in vivo* concentration difference compensates for the difference in MIC and the concentration at which the peptoids and peptides aggregated ribosomes. Peptoid **2** was used as a negative control, as it has same charge (+4) as other peptoids and a similar structure, but is less hydrophobic and is inactive against bacteria. Peptoid **2** was unable to aggregate the bacterial ribosomes, which implies that the aggregation of ribosomes is not merely due to electrostatic interactions between positively charged peptoid and negatively charged ribosomes. We hypothesize that peptoids also would aggregate purified mammalian ribosomes in culture (not yet tested), but the *in vivo* selectivity would be driven by the preferential attraction of peptoids for negatively charged bacterial membrane over zwitterionic outer mammalian membrane, as well as by the fact that in mammalian cells, many ribosomes are anchored and scaffolded on endoplasmic reticulum (ER) membranes and, within mammalian mitochondria, within the membranes of the cristae.

## Conclusions

Unlike many clinically used antibiotics, which inhibit a specific protein target, most AMP mechanisms do not involve stereospecific interactions with receptors or enzymes, since enantiomeric versions typically possess activities congruent to the native peptides^17,63^. Consequently, AMP and ampetoid mechanisms must rely on “pattern recognition”-type biophysical interactions that—based on the cationicity and amphipathicity shared by nearly all of these compounds—are mediated by electrostatics and hydrophobic interactions. Many AMPs have been shown to translocate into the cytoplasm^26,49–51,64^ and some have demonstrated intracellular granulation effects^52,53^; furthermore, there is increasing evidence that AMPs and cell-penetrating peptides are closely related, if not indistinguishable, classes of molecules^65^. Some AMPs, including LL-37 and human neutrophil peptide-1 (HNP-1), also exhibit anticancer activities and AMPs are being investigated as a source for anticancer therapeutics^66^. HNP-1 is reported to induce chromatin condensation as part of its mechanism of action against solid tumor cells^67^. We have previously reported peptoid **1** to have potent anticancer activity both *in vitro* and *in vivo* ^68^ and this activity may be linked to the ability of peptoid **1** to bind DNA. In light of these considerations, any complete paradigm of AMP mechanism must not focus solely on membrane interactions without also considering intracellular effects; the ability of these molecules to penetrate through membranes means they will inevitably interact with cytoplasmic components. What has not been appreciated until now, is the extent of devastating physical transformations of intracellular biomass distribution that these interactions effect.

We propose that AMPs and ampetoids effect intracellular aggregation of biomolecules, causing intrabacterial polyanions, which also have substantial aromatic hydrophobic characteristics in the RNA and DNA bases, to flocculate, a phenomenon readily visible in Fig. 4. There are many ubiquitous polyanions in the bacterial proteome other than nucleic acids and ribosomes^69^ including homologs of actin^70^. It has been hypothesized that this “network” of polyanions plays a vital role in many cellular processes^69^. By this novel mechanism, the polyanionic network is coalesced by AMPs and ampetoids, and thus the spatial organization, free diffusion, and accessibility of cytoplasmic components are all simultaneously disrupted. In this sense, AMPs and ampetoids do not so much “target” cytoplasmic species as much as they interfere with the spatial organization of the intrabacterial compartment non-specifically. We hypothesize that these changes are damaging enough to inhibit growth, and in many cases, to kill bacteria altogether.

Despite the importance of intracellular effects to the mechanisms of AMPs and their mimics, it must be emphasized that studies of membrane permeabilization remain integral to a comprehensive understanding of these molecules. However, researchers have ruled out an osmotic lysis mechanism for AMPs^40,41^, and depolarization in itself is not lethal—many AMPs cause maximal depolarization at sub-inhibitory concentrations^8,27–30^ and the action of many respiratory uncouplers is reversible^71^. It follows that lipid perturbation and pore formation are essential to the antibacterial mechanisms of many of these oligomers only insofar as they facilitate their translocation into the cytoplasm^27^. In this sense, membrane interactions should be closely related but not correlated to antibacterial activity—the true correlation should exist between antibacterial activity and the sum of abilities of a given peptide to cross (and in some cases disrupt) the membrane *and* to interact with intracellular targets.

## Material and Methods

### Materials

Peptoids were synthesized on solid phase on an ABI 433 peptide synthesis robot, as described previously^17^. Peptides (pexiganan, melittin) were synthesized using standard Fmoc chemistry. Mass spectrometry was used to confirm the molecular weight of the purified product. LL-37 was generously donated by Dr. David Gidalevitz at Illinois Institute of Technology. Chicken fowlicidin-1 (also called cathelicidin-1) was generously donated by Dr. Edwin Veldhuizen at Utrecht University, Utrecht, the Netherlands.

#### Antibacterial assays

MICs were determined according to CLSI M7-A6 protocols in a 96-well microtiter plate. In test wells, 50 μL bacterial inoculum (1 × 10^6^ CFU/ml) in cation-adjusted Mueller-Hinton broth (CAMHB) was added to 50 μL peptoid solution in CAMHB (prepared by 1:2 serial dilutions). Positive controls contained 50 μL inoculum and 50 μL CAMHB without peptoid. The MIC was defined as the lowest concentration of peptoid that completely inhibited bacterial growth after incubation at 35 °C for 16 h. MIC values reported were reproducible between three independent experimental replicates, each consisting of two parallel trials.

#### Hemolysis assays

Erythrocytes were isolated from freshly drawn, heparanized human blood and resuspended to 20 vol% in PBS (pH 7.4). In a 96-well microtiter plate, 100 μL erythrocyte suspension was added to 100 μL peptoid solution in PBS (prepared by 1:2 serial dilutions), or 100 μL PBS in the case of negative controls. 100% hemolysis wells contained 100 μL blood cell suspension with 100 μL 0.2 vol% Triton X-100. The plate was incubated for 1 h at 37 °C, then each well was diluted with 150 μL PBS. The plate was then centrifuged at 1200 g for 15 min, 100 μL of the supernatant from each well transferred to a fresh microtiter plate, and A_350_ measured. Percent hemolysis was determined as (A − A_0_)/(A_tota_l − A_0_) × 100, where A is the absorbance of the test well, A_0_ the absorbance of the negative controls, and A_total_ the absorbance of 100% hemolysis wells, all at 350 nm. Hemolysis data was reproducible between three independent experimental replicates each with at least 3 parallel trials, except for LL-37 and fowlicidin-1 for which two independent experimental replicates with 2 parallel trials were run due to limited compound availability.

#### Calcein leakage

POPE/POPG (7:3) or POPC/cholesterol (2:1) in chloroform were dried under N_2_ and lyophilized overnight. The resulting film was hydrated at 37 °C for 1 hour in 70 mM calcein, 10 mM Tris buffer, pH 7.4 to a final lipid concentration of 20 mM, then vortexed until evenly suspended. The lipid suspension was subjected to five freeze-thaw cycles (liquid N_2_/37 °C water bath) then loaded into a syringe and extruded through two stacked 100 nm polycarbonate membranes for at least 15 passes to create LUVs. Free calcein was separated from LUVs using a Sephadex G-50 column with 10 mM Tris, 100 mM NaCl, pH 7.4 running buffer. The lipid concentration of the LUV fraction was determined using a phosphorus assay adapted from Bartlett^72^. To measure leakage, LUV stock was added to 10 mM Tris, 100 mM NaCl, pH 7.4 to a final concentration of 50 µM lipids in a 1.5 mL rectangular quartz fluorescence cuvette (λ_ex_ = 490 nm, λ_em_ = 520 nm, 1 mm bandwidth), and gently aspirated to mix. After the signal stabilized, aqueous peptoid stock was added to the desired final concentration, and gently aspirated to mix. After 5 minutes, 5 µL of aqueous 10% Triton X-100 was added and aspirated to get the 100% leakage value for that run. Percent dequenching at each concentration was calculated as 100 × (F − F_0_)/(F_T_ − F_0_), where F was the fluorescence 5 minutes after the addition of the peptide/peptoid, F_0_ was the initial fluorescence (before peptide addition), and F_T_ was the total fluorescence (after Triton X-100 addition).

#### Membrane depolarization

*E. coli* ATCC 35218 bacteria, grown overnight at 35 °C in Mueller-Hinton broth, were resuspended in 5 mM HEPES, 20 mM glucose, pH 7.4, and diluted in the same buffer to an OD_590_ of 0.05. The bacterial suspension was divided into 1.5 mL aliquots, each of which were individually treated with EDTA (final concentration = 0.2 mM, to facilitate penetration of dye into the bacteria) and diSC3-5 in DMSO (final concentration = 0.2 µM). Individual treatment of each aliquot was necessary because the fluorescence response of the diSC3-5 was found to vary with the amount of time it was in contact with the bacteria (data not shown). One hour after dye treatment, each dyed bacterial suspension was loaded into a 1.5 mL rectangular quartz fluorescence cuvette (λ_ex_ = 622 nm, λ_em_ = 670 nm, 1 mm bandwidth). After recording the baseline fluorescence, aqueous peptide/peptoid stock was added to the desired final concentration and aspirated gently to mix. Five minutes later, melittin was added to a final concentration of 20 µM to achieve maximal depolarization. Percent depolarization was calculated analogously to that in calcein leakage experiments.

#### Scanning electron microscopy

Overnight cultures of *E. coli* ATCC 35218 were centrifuged for 10 min and 2500 × g at room temperature, then resuspended in PBS. The bacterial suspensions were diluted in PBS to an OD590 < 0.10. Bacterial concentrations of the diluted suspensions were calculated from their OD590 values using optical density curves derived from pre-determined concentrations. The diluted suspensions were then diluted further in PBS to give 5 × 10^5^ CFU/mL test suspensions, which is identical to the final concentration of bacteria in an MIC assay. One mL aliquots of the test suspensions were combined with aqueous peptide/peptoid stocks (typically ~10 mg/mL) to a final concentration of 10 μM. After 1 hr, small volumes of each sample were serially diluted, plated on growth agar, and incubated overnight for counting. The remainder of each sample was collected on Isopore polycarbonate membrane filters (Millipore) with 0.2 μm pores. Using microwave assistance, the bacteria-coated filters were fixed (1% glutaraldehyde, 1% paraformaldehyde, 50 mM cacodylate buffer, pH 7.4; 2 min @ 82 W, then 2 min @ 216 W), rinsed in 50 mM cacodylate buffer, pH 7.4 (2X, 1 min @ 82 W), and dehydrated in an ascending ethanol series (30%, 50%, 70%, 90%, and 100%; 2X each step, 40 sec @ 82 W). The dehydrated samples were dried at the critical point in CO2, mounted on stubs with carbon tape, sputter coated with Au/Pd to a thickness of 8 nm, then imaged on a Hitachi S-4800-II SEM at 1.0 kV with a working distance of 3 mm.

#### Transmission electron microscopy

*E. coli* ATCC 35218, grown overnight at 35 °C in Mueller-Hinton broth (MHB), were resuspended in 1X (150 mM) PBS, pH 7.4, and diluted to a concentration of 1 × 10^8^ CFU/mL. Aqueous peptide/peptoid stock solutions were added to 1 mL aliquots of the suspension to the desired final concentration. After 1 hour, small volumes of each sample were serially diluted, plated, and incubated overnight at 35 °C, to be counted the following day. The remaining volume of each sample was centrifuged, and the pellet resuspended and enrobed in 5–10 µL of 2% low-melting agarose (gelling temperature 25° ± 5 °C) then drawn up into the tip of a Pasteur pipette. The solidified rods were extruded into fixative (2% glutaraldehyde, 2% paraformaldehyde, 50 mM PBS, pH 7.4). Using microwave assistance, the enrobed bacteria were fixed (2 min @ 82 W, then 2 min @ 216 W), rinsed in 50 mM PBS, pH 7.4 (2X, 1 min @ 82 W), osmicated in 1% aqueous OsO_4_ (2 min @ 82 W), rinsed in H_2_O (2X, 1 min @ 82 W), dehydrated in an ascending acetone series (30%, 50%, 70%, 90%, and 100%; 2X each step, 40 sec @ 82 W), infiltrated in 25%, 50%, 75%, and 100% Eponate 12 resin in acetone (1X each step, 3 min @ 82 W), and embedded in the same resin. 50 nm thin sections of embedded samples were cut using a diamond knife, stained for 5 min in 5% aqueous uranyl acetate, then rinsed in H_2_O. Sections were examined in a JEOL 1230 TEM. Experimental conditions were each repeated in at least two independent trials. For each replicate of each sample, bacteria across the thin section were first surveyed to determine morphological characteristics representative of that sample, then images were captured of at least five bacteria displaying those morphologies with an emphasis on clean (i.e., orthogonal) transverse and/or longitudinal sections. Each image shown is thus representative of the ultrastructural characteristics observed in bacteria throughout a given sample and between replicates. In each TEM experiment, the ultrastructure of the untreated control was confirmed to be consistent with that from every other experiment.

### Soft X-ray tomography

*E. coli* (ATCC 35218) were inoculated from an overnight culture and grown in MHB at 37 °C for approximately 2 hours to the exponential growth phase (OD ~0.4). Cells were taken directly from their culture flasks, incubated with peptoid for 20 minutes, transferred by pipette into a thin-walled glass capillary tube, and then flash-frozen in liquid nitrogen. No additional staining procedures were used. Imaging was performed with the specimens in an atmosphere of liquid nitrogen cooled helium gas at all times. X-rays were collected using the XM-2 soft X-ray microscope operated by the National Center for X-ray Tomography (http://ncxt.lbl.gov) at the Advanced Light Source (http://www.als.lbl.gov) of Lawrence Berkeley National Laboratory (LBNL). XM-2 is equipped with Fresnel zone plate based condenser and objective lenses (made by the Center for X-ray Optics, LBNL) and is specifically designed to investigate biological samples at cryogenic temperatures.

#### DNA retardation

DNA retardation was analyzed by gel electrophoresis. Peptoid/peptide-DNA complexes were formed by adding 10 µL of peptide/peptoid solution of varying concentrations to 10 µL DNA solution (EGFP-Luc plasmid in Tris-buffered saline (TBS) buffer), and the resulting solution was mixed by gentle aspiration. Complexes were incubated for 20 min at room temperature. After complex formation, 20 µL of each sample were loaded onto a 1–2% agarose gel in Tris/borate/EDTA (TBE) buffer, which ran at 120 V for 15 min. DNA bands on the gel were visualized under UV light by ethidium bromide staining.

### Kinetics of antibacterial activity

Bioluminescent *P. aeruginosa* ^73^ was grown at 37 °C in Luria-Bertani (LB) broth. The kinetics of antibacterial activity was determined at 12.5 µM at 37 °C in a 96-well black, clear bottom microtiter plates, such that bioluminescence could be used to indicate cell viability^74^. In a 96-well plate, 50 µL of *P. aeruginosa* solution (1 × 10^6^ CFU/mL) was added to 50 µL of antimicrobial solution, and the bioluminescence was measured using an IVIS imaging system (a Xenogen product from Caliper Life Sciences, Hopkinton, MA) at 10 min intervals for 2 h. The reported values are the average of three independent experiments with two replicates each.

### Bacterial plating of *P. aeruginosa*

In a 96-well plate, bacterial samples were treated with 12.5 µM antimicrobials in LB at 37 °C for 10 min. The bacterial counts were determined by plating serial dilutions of samples on LB plates and counting bacterial colonies. The reported values are the average of three replicates.

### Aggregation of bacterial ribosomes

In a microcentrifuge tube, 5 µl of 1 µM purified bacterial ribosome solution (~40X diluted (v/v) from their intracellular concentration in bacterial cytosol)^62^ was incubated with 5 µl of antimicrobial solution in a concentration range of 0–800 µM for 10 min. The absorbance of the solution was measured by UV-Vis spectroscopy at λ = 550 nm by a spectrophotometer (ND-1000, Thermo Scientific, Wilmington, DE). The values are reported as the average of three independent experiments with three replicates each.

## Electronic supplementary material

Supplementary Information
