# Supplementary material for: Intracellular biomass flocculation as a key mechanism of rapid bacterial killing by cationic, amphipathic antimicrobial peptides and peptoids
Source: Sci Rep. 2017 Dec 1;7:16718. doi: 10.1038/s41598-017-16180-0 (PMC5711933; doi:10.1038/s41598-017-16180-0)
Supplement: Supplementary file 1 — Supplementary Information [file 41598_2017_16180_MOESM1_ESM.pdf]

Supplementary Information

Intracellular biomass flocculation as a key mechanism of rapid bacterial killing by cationic, amphipathic antimicrobial peptides and peptoids

Nathaniel P. Chongsiriwatana[1], Jennifer S. Lin[2], Rinki Kapoor[3], Modi Wetzler[2], Jennifer A. C. Rea[1], Maruti K. Didwania[2], Christopher H. Contag[4] and Annelise E. Barron[2]*

[1]Department of Chemical and Biological Engineering, Northwestern University, Evanston, Illinois; [2]Department of Bioengineering, [3]Biophysics Program, and [4]Departments of Microbiology and Immunology, Pediatrics, and Radiology, Stanford University, Stanford, California

*Annelise E. Barron

Associate Professor

Department of Bioengineering, Stanford University

Shriram Center, Room 229

443 Via Ortega

Stanford, CA 94305

Phone: 650-946-8039

E-mail: aebarron@stanford.edu

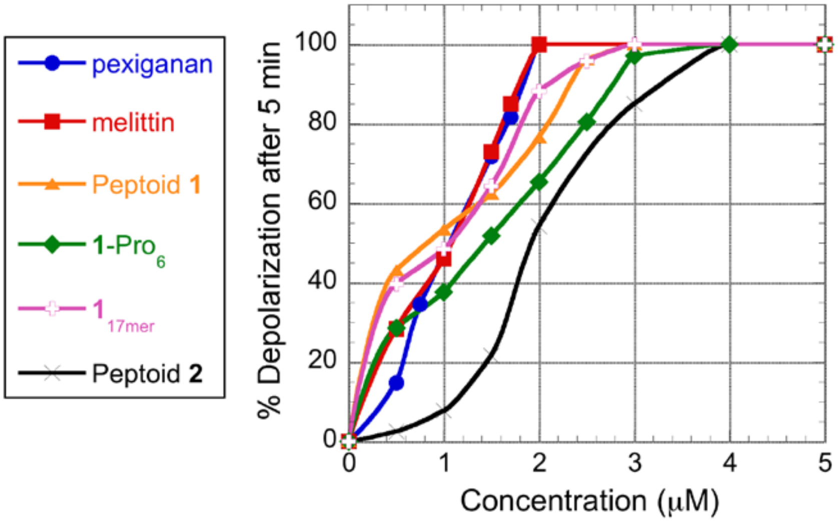

Figure S1. Membrane depolarization of *B. subtilis* ATCC 6633 after 5-min. treatment, as monitored by diSC3-5 fluorescence. The data are representative of 3 independent experiments.

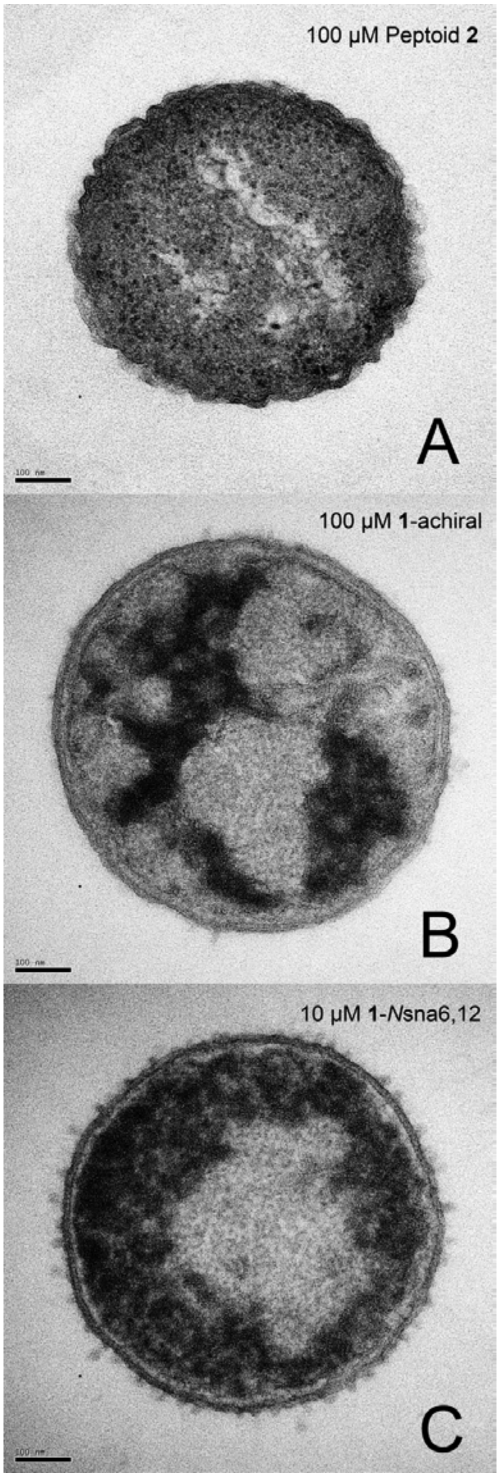

Figure S2. Transmission electron micrographs of *E. coli* bacteria treated with **(A)** 100 µM peptoid **2**, **(B)** 100 µM $\mathbf{1}_{achiral}$, or **(C)** 10 µM **1**-Nsna$_{6,12}$ for 1 hour.

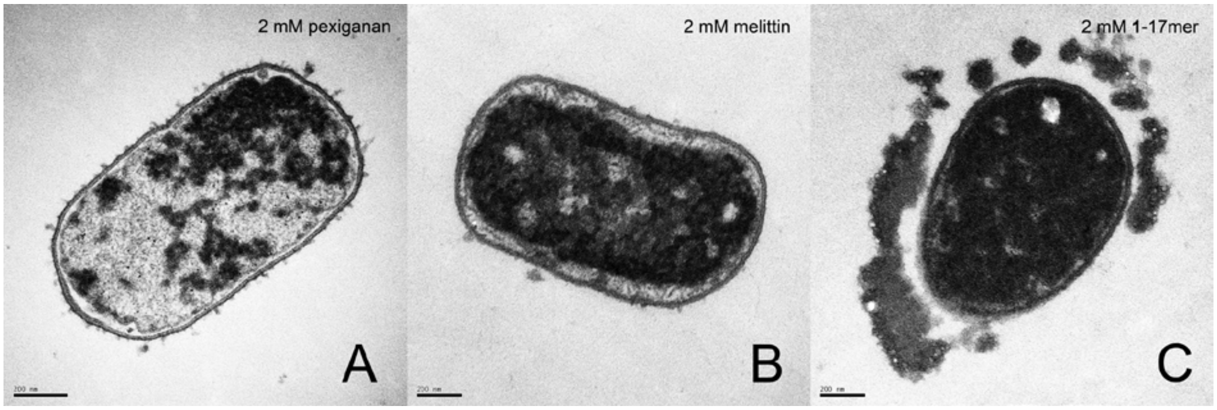

Figure S3. Transmission electron micrographs of longitudinal sections of *E. coli* bacteria treated with 2 mM **(A)** pexiganan, **(B)** melittin, and **(C)** peptoid $\mathbf{1}_{17mer}$.

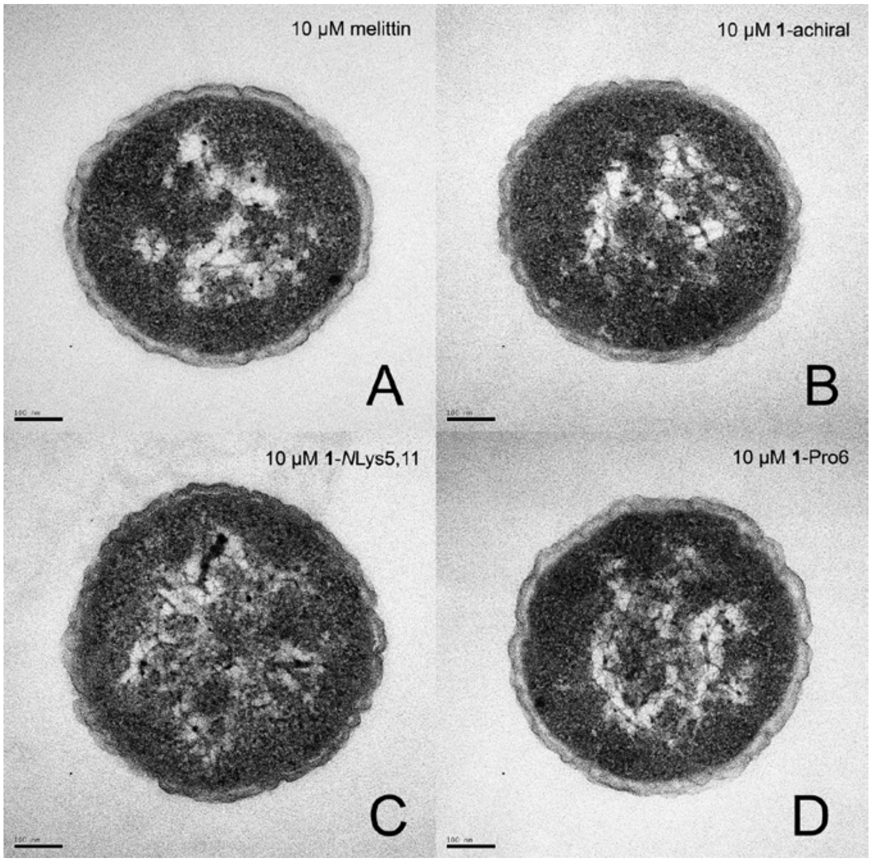

Figure S4. Transmission electron micrographs showing *E. coli* treated with sub-lethal concentrations (i.e. concentrations which killed none of the bacteria in each sample) of **(A)** 10 µM melittin, **(B)** 10 µM $\mathbf{1}_{achiral}$, **(C)** 10 µM **1**-*N*$Lys_{5,11}$, and **(D)** 10 µM **1**-Pro_6.

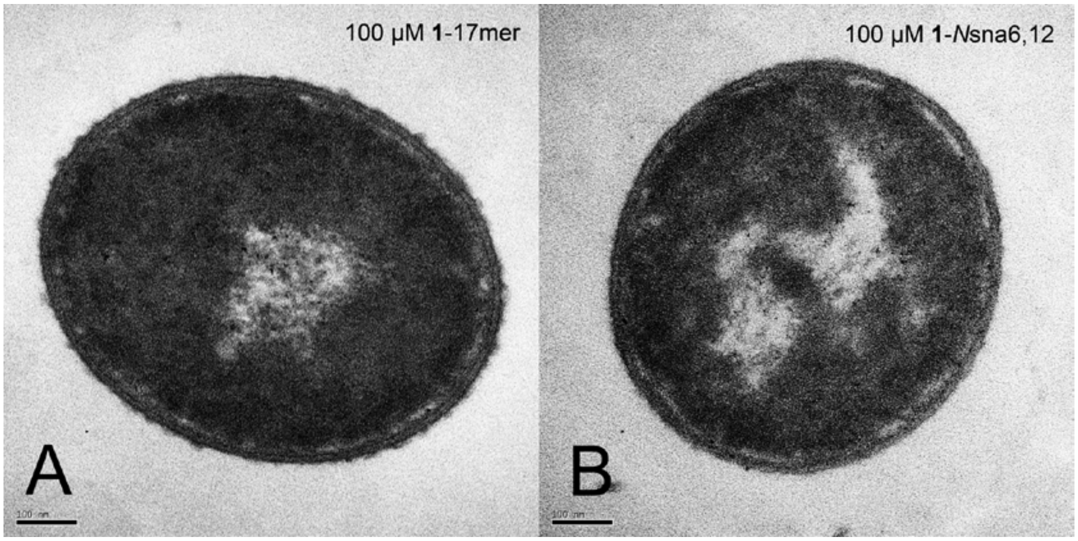

Figure S5. Transmission electron micrographs of *E. coli* treated with 100 μM peptoids **(A) 1**$_{17mer}$ and **(B) 1**-*N*sna$_{6,12}$ for 1 hour.

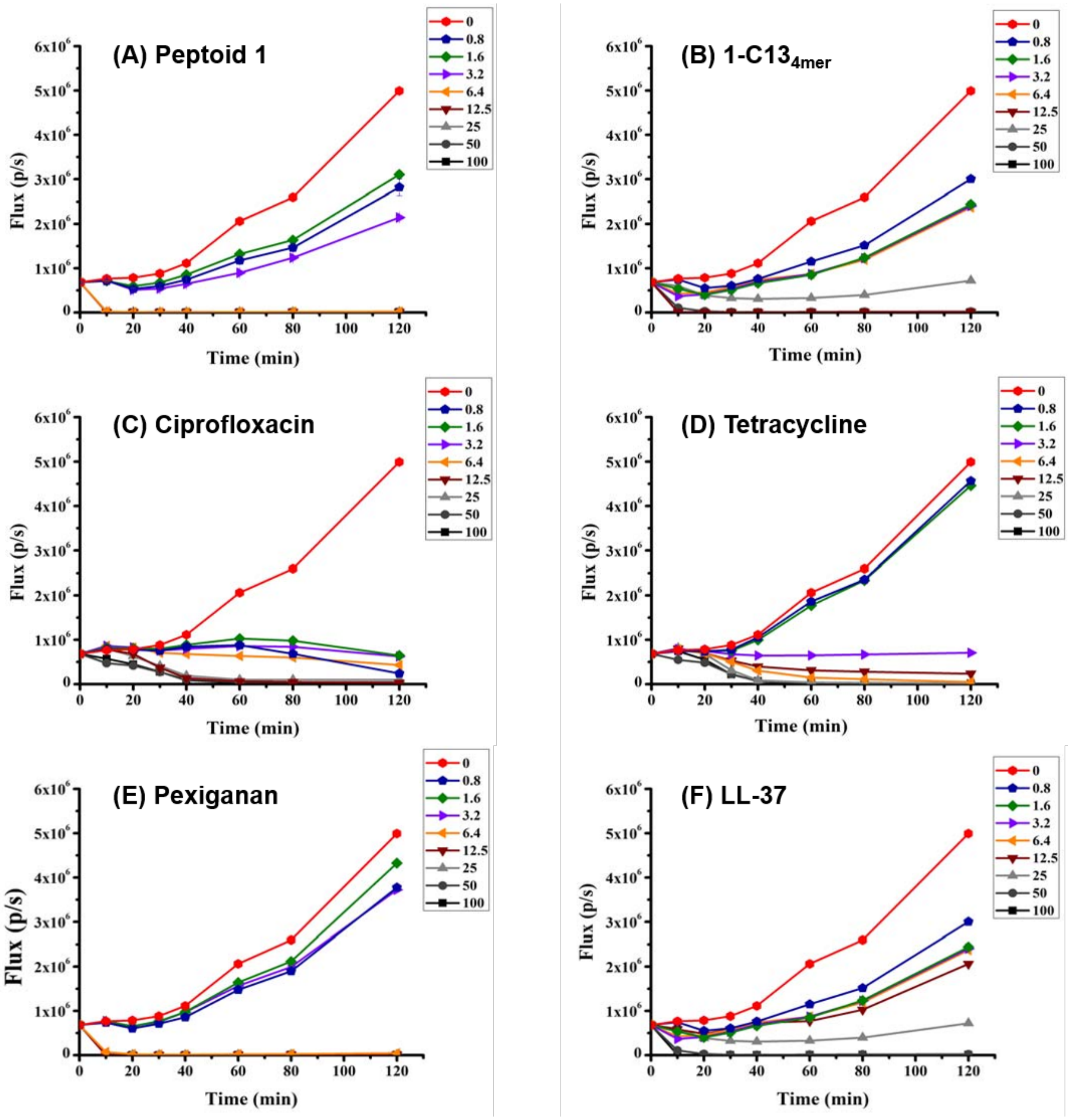

Figure S6. Kinetics of antibacterial activities of (A) Peptoid 1, (B) 1-C13$_{4mer}$, (C) Ciprofloxacin, (D) Tetracycline, (E) Pexiganan, and (F) LL-37 against bioluminescent *P. aeruginosa* from 0 - 100 μM in LB at 37 ºC. Flux represents number of photons emitted by bacteria per second. Reported values are average of three independent experiments with two replicates each.

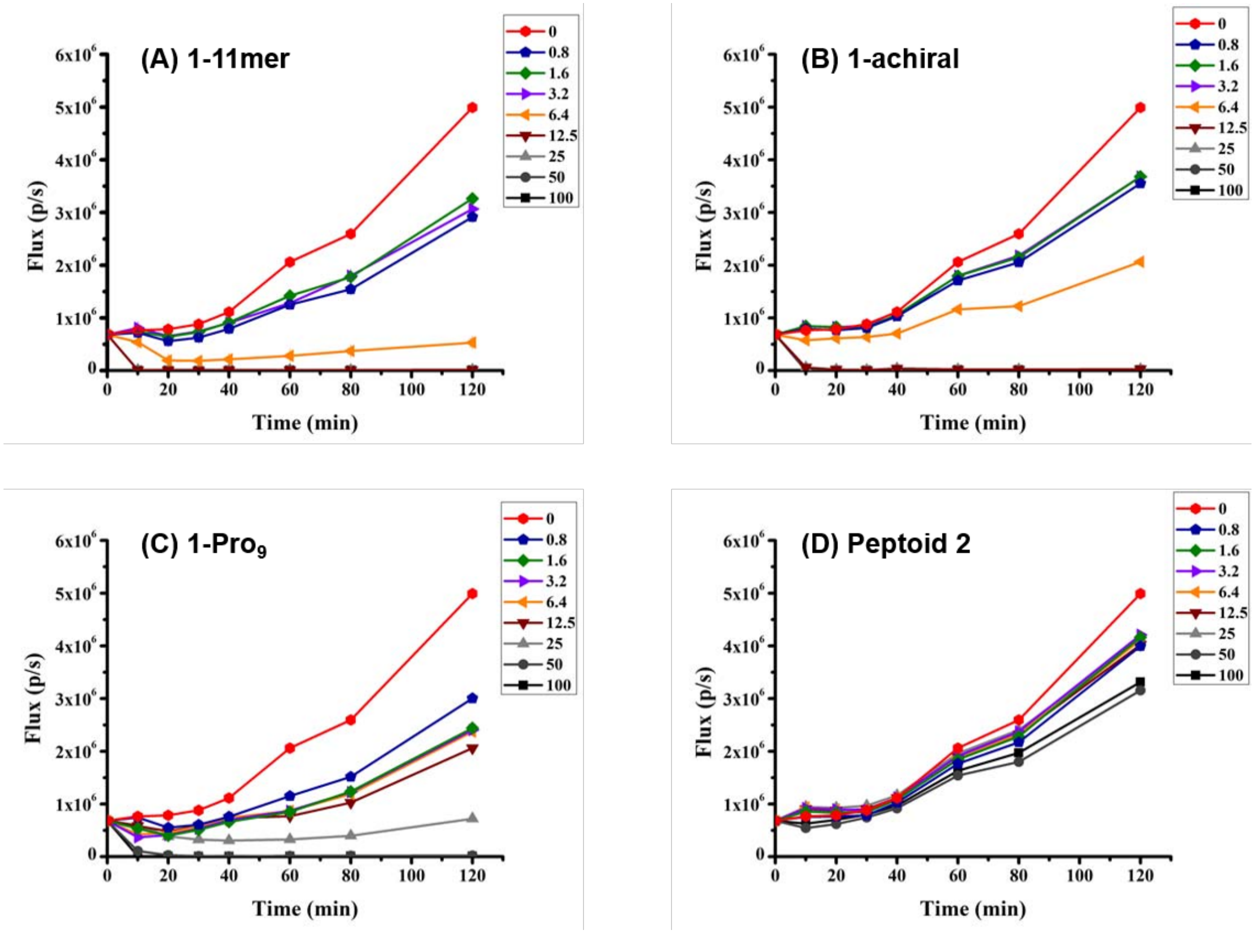

Figure S7. Kinetics of antibacterial activities of (A) 1-11mer, (B) 1-achiral, (C) 1-Pro$_9$, and (D) Peptoid 2 against bioluminescent *P. aeruginosa* from 0 - 100 μM in LB at 37 ºC. Flux represents number of photons emitted by bacteria per second. Reported values are average of three independent experiments with two replicates each.
